# Association between usual alcohol consumption and risk of falls in middle-aged and older Chinese adults

**DOI:** 10.1186/s12877-022-03429-1

**Published:** 2022-09-14

**Authors:** Yue Sun, Baiyang Zhang, Qiang Yao, Yao Ma, Yidie Lin, Minghan Xu, Meijing Hu, Jingjing Hao, Min Jiang, Changjian Qiu, Cairong Zhu

**Affiliations:** 1 Department of Epidemiology and Health Statistics, West China School of Public Health and West China Fourth Hospital, Sichuan University, Chengdu, China; 2 Mental Health Center, West China Hospital of Sichuan University, No. 37 Guo Xue Xiang, Chengdu, 610041 China; 3 West China-PUMC C.C. Chen Institute of Health, Sichuan University, Chengdu, China

**Keywords:** Middle-aged and older adults, Alcohol consumption, Falls, Longitudinal

## Abstract

**Background:**

Previous studies exploring usual alcohol consumption and falls risk were scarce in China. In addition, the dose–response relationship has not been explored so far. This study aims to estimate the association between usual alcohol consumption and risk of falls among middle-aged and older Chinese adults based on data from the China Health and Retirement Longitudinal Study (CHARLS), which is representative of the population of the entire country.

**Methods:**

Baseline survey data in 2015 and follow-up data in 2018 in CHARLS were utilized. Alcohol consumption was calculated in grams per day (gr/day) according to self-reported drinking data and categorized accordingly to The Dietary Guidelines for Chinese Residents (DGC) 2016. Fall was obtained from self-reported information. Multivariable logistic regression analyses were performed to estimate the association of usual alcohol consumption with risk of falling. The dose–response relationship was also explored using restricted cubic splines.

**Results:**

A total of 12,910 middle-aged and older participants were included from the CHARLS 2015, of which 11,667 were followed up in 2018. We found that former, moderate, and excessive drinkers were at higher fall risk compared to never drinkers (former: OR, 1.24; 95% CI, 1.05–1.46; moderate: OR, 1.22; 95% CI, 1.06–1.41; excessive: OR, 1.36; 95% CI, 1.15–1.61) in the longitudinal analysis. Similarly, individuals with moderate and excessive alcohol consumption were at increased risk of falling in the cross-sectional analysis (moderate: OR, 1.18; 95% CI, 1.02–1.37; excessive: OR, 1.32; 95% CI, 1.11,1.57). No significant increased risk of falls was found for former drinkers (former: OR, 1.13; 95% CI, 0.96–1.34). We observed a significant non-linear relationship.

**Conclusions:**

Our study suggests that usual alcohol consumption was associated with a higher risk of falls, highlighting the key role of alcohol intake on the fall risk, which needed consideration in developing intervention and prevention strategies for reducing falls among middle-aged and older Chinese adults.

**Supplementary Information:**

The online version contains supplementary material available at 10.1186/s12877-022-03429-1.

## Introduction

Falls pose a major health risk especially for middle-aged and older adults, given their high incidence, mortality rates, and healthcare costs. According to the World Health Organization, there are about 684,000 people die of fall injuries annually, with over 80% in low-income and middle-income countries among them [1]. Ye et al. found that the incidence rate of falls rises greatly among older participants in mainland China between 1990 and 2019, which is based on the data from the Global Burden of Disease Study 2019 [2].

Numerous studies have explored the relationship between usual alcohol consumption and falls, but the results are inconsistent [3–10]. Some suggested that usual alcohol consumption is a risk factor for falls [3–6], while others found inverse or null associations [7–10]. Most studies have been limited by the cross-sectional design [3, 5–7, 9] or the case–control design [8], because of which, the sequence of the exposure and outcome cannot be guaranteed [11]. Potential selection bias might also limit studies with case–control design, which contributed to the lack of representativeness of the study samples. Furthermore, insufficient follow-up period, small sample size, and inadequate covariates being considered might have limited remaining prospective studies [4, 10]. It should be noted that alcohol consumption is increasing faster in China than in other parts of the world [12, 13]. However, the majority of previous studies were conducted in developed countries, for instance, the United States [4, 5, 7, 8, 10], Australia [14], Spain [3], Sweden [15], etc. Relevant evidence in developing countries, such as China, remained scarce. In addition, it was reported that accumulated alcohol intake can lead to peripheral neuropathy [16], thereby damaging bone health [17, 18], which is a crucial contributing factor to falls. Besides, excessive usual alcohol intake increases the risk of cognitive decline and the risk of dementia, both of which have also been proved to have considerable effects on falls [19–22]. Based on the above statements, we propose the hypothesis that usual alcohol consumption is a risk factor for falls regardless of the dose. In addition, previous studies only analyzed alcohol consumption as a binary variable (e.g., drunk or not) or a multi-categorical variable (e.g., never drinkers, moderate drinkers, and excessive drinkers) [3–10]. To date, alcohol consumption studies in falls lack evaluation of any dose–response effects. Because categorization might obscure potentially vital discrepancies in fall risk across groups of alcohol intake [23], we are supposed to quantify whether increasing alcohol consumption leads to an increased risk of falls in Chinese populations.

In summary, we aimed to estimate the association between usual alcohol consumption and risk of falling both in cross-sectional and longitudinal analysis, with consideration of potential dose–response relationship, among middle-aged and older Chinese adults using data from the China Health and Retirement Longitudinal Study (CHARLS), in order to provide a reference for falls prevention and establishment of the intervention measures.

## Methods

### Data

Nationwide representative data was acquired from China Health and Retirement Longitudinal Study (CHARLS), an up-to-date cohort study implemented by the National School for Development (IRB00001052-11,015) [24]. Participants are followed in 2013, 2015, and 2018 using computer-assisted interviews with the national baseline survey started in 2011. Multistage probability sampling was used to select family samples over 45 years old. A total of 28 provinces were chosen in CHARLS, from where 435 rural or urban communities were selected, for which the whole cohort could involve a wide range of areas including various economic conditions, and could have the advantages of large sample size and strong representation. CHARLS had a high response rate of 80.5%, which further enhances the reliability and representativeness of the study. For non-response individuals, various reasons are also recorded in detail, including 8.8% that refused, 8.2% that disable to connect, and 2% for other causes. All available data in CHARLS is obtained from three parts: (1) face-to-face interviews administered by trained investigators in the sampled household using a well-designed questionnaire; (2) physical examinations performed using formal instruments and (3) laboratory blood tests at specific healthcare organizations locally. For the first part, researchers designed a questionnaire with extremely abundant content and included plenty of health-related issues. Investigators previously received collective training to collect information with the computer-assisted interview to ensure accuracy. More detailed information about the CHARLS can be found in previous literature. All data in the present study were acquired from the CHARLS that was carried out in 2015 and 2018. A total of 12,910 middle-aged and older participants were included in the CHARLS 2015, of which 11,667 participants were followed up in 2018 (Table 1).

**Table 1:** Characteristics of 11,667 participants according to baseline alcohol consumption

	Total, n (%)	Never-drinkers, n (%)	Former-drinkers, n (%)	Moderate-drinkers, n (%)	Excessive-drinkers, n (%)
Total, n (%)	11,667(100.0)	7588(100.0)	1044(100.0)	1764(100.0)	1271(100.0)
Age, years	61.0 ± 9.2	60.8 ± 9.4	64.0 ± 9.3	60.0 ± 8.9	61.2 ± 8.6
Gender, n (%)					
Female	6296(54.0)	5505(72.6)	261(25.0)	421(23.9)	109(8.6)
Male	5371(46.0)	2083(27.5)	783(75.0)	1343(76.1)	1162(91.4)
Residence, n (%)					
Rural	4170(35.7)	2737(36.1)	383(36.7)	635(36.0)	415(32.7)
Urban	7497(64.3)	4851(63.9)	661(63.3)	1129(64.0)	856(67.4)
Education, n (%)					
No formal education	3053(26.2)	2343(30.9)	232(22.2)	297(16.8)	181(14.2)
Sishu/Homeschool/Elementary school	4937(42.3)	3127(41.2)	460(44.1)	739(41.9)	611(48.1)
Middle school and above	3677(31.5)	2118(27.9)	352(33.7)	728(41.3)	479(37.7)
Marital status, n (%)					
Cohabited	9689(83.1)	6208(81.8)	864(82.8)	1509(85.5)	1108(87.2)
Living alone	1978(17.0)	1380(18.2)	180(17.2)	255(14.5)	163(12.8)
Smoke, n (%)					
No	8476(72.7)	6300(83.0)	658(63.0)	1023(58.0)	495(39.0)
Yes	3191(27.4)	1288(17.0)	386(37.0)	741(42.0)	776(61.1)
Sleep duration, n (%)					
< 7 h	2071(17.8)	1333(17.6)	177(17.0)	325(18.4)	236(18.6)
7–8 h	6172(52.9)	4025(53.0)	563(54.0)	931(52.8)	653(51.4)
≥ 8 h	3424(29.4)	2230(29.4)	304(29.1)	508(28.8)	382(30.1)
Daytime napping, n (%)					
0 min	1765(15.1)	1180(15.6)	150(14.4)	269(15.3)	166(13.1)
0–30 min	4991(42.8)	3426(45.2)	417(39.9)	694(39.3)	454(35.7)
30–60 min	2734(23.4)	1694(22.3)	263(25.2)	426(24.2)	351(27.6)
> 60 min	2177(18.7)	1288(17.0)	214(20.5)	375(21.3)	300(23.6)
Body mass index, n (%)					
Underweight	663(5.7)	437(5.8)	64(6.1)	96(5.4)	66(5.2)
Normal	5623(48.2)	3485(45.9)	519(49.7)	912(51.7)	707(55.6)
Overweight	3821(32.8)	2526(33.3)	342(32.8)	575(32.6)	378(29.7)
Obesity	1560(13.4)	1140(15.0)	119(11.4)	181(10.3)	120(9.4)
Depression, n (%)					
No	7188(61.6)	4470(58.9)	629(60.3)	1178(66.8)	911(71.7)
Yes	4479(38.4)	3118(41.1)	415(39.8)	586(33.2)	360(28.3)
IADL ^a^, n (%)					
Independent	10,165(87.1)	6521(85.9)	858(82.2)	1604(90.9)	1182(93.0)
Dependent	1502(12.9)	1067(14.1)	186(17.8)	160(9.1)	89(7.0)
Pain, n (%)					
No	8023(68.8)	5035(66.4)	681(65.2)	1290(73.1)	1017(80.0)
Yes	3644(31.2)	2553(33.7)	363(34.8)	474(26.9)	254(20.0)
Handgrip strength, n (%)					
High	7617(65.3)	4927(64.9)	606(58.1)	1205(68.3)	879(69.2)
Low	4050(34.7)	2661(35.1)	438(42.0)	559(31.7)	392(30.8)
Comorbidities, n (%)					
0	3835(32.9)	2496(32.9)	236(22.6)	618(35.0)	485(38.2)
1	3358(28.8)	2197(29.0)	271(26.0)	507(28.7)	383(30.1)
≥ 2	4474(38.4)	2895(38.2)	537(51.4)	639(36.2)	403(31.7)
Fall, n (%)					
No	9267(79.4)	6014(79.3)	806(77.2)	1421(80.6)	1026(80.7)
Yes	2400(20.6)	1574(20.7)	238(22.8)	343(19.4)	245(19.3)
Alcohol consumption, n (%)					
Never-drinkers	7588(65.0)	7588(100.0)	-	-	-
Former-drinkers	1044(9.0)	-	1044(100.0)	-	-
Moderate-drinkers	1764(15.1)	-	-	1764(100.0)	-
Excessive-drinkers	1271(10.9)	-	-	-	1271(100.0)

^a^ *Abbreviations*: *IADL* instrumental activities of daily living

### Measurements

#### Alcohol consumption

Never drinkers and former drinkers were identified based on a ‘no’ response to the question “Did you drink any alcoholic beverages, such as beer, wine, or liquor in the past year? (If ‘yes’) How often?”. Then, among the participants who answered ‘no’, those who indicated that they had never drunk alcoholic beverages or had never drunk more than once a month before the last year were classified as ‘never drinkers’, and those who had drunk more than once a month before the last year were classified as ‘former drinkers’.

Those who indicated they drank more than monthly in the last year were identified as ‘current drinkers’. For all current drinkers, follow-up questions were asked about their drinking frequency of liquor, beer, and wine and their drinking capacity for each kind of drinking. The Dietary Guidelines for Chinese Residents (DGC) 2016 [25] defined moderate alcohol consumption as up to 25 g of ethanol per day for men and 15 g for women. Therefore, moderate drinkers were defined as < 25 g of ethanol per day for men and < 15 g for women. Excessive drinkers were further defined as ≥ 25 g of ethanol per day for men and ≥ 15 g for women.

#### Fall

Fall is defined as an unexpected event in which the participants come to rest on the ground, floor, or lower level [26]. Baseline fall was classified into two categories based on the ‘yes’ or ‘no’ answer to the question “Have you fallen down since 2013 (or in the last 2 years)?” in the CHARLS 2015. Similarly, in the CHARLS 2018, fall was classified into two categories based on the ‘yes’ or ‘no’ answer to the question “Have you fallen down since 2015 (or in the last 3 years)?”.

#### Covariates

Based on previous literature and relevant expertise, we selected the following variables as covariates. Demographic characteristics included age, gender, residence, educational level and marital status. Health-related behaviors included Smoking status, sleep duration and daytime napping. Health status included body mass index (BMI), handgrip strength, pain, instrumental activities of daily living (IADL), comorbidities and depression. Age is a continuous variable. The residence was categorized as rural or urban. Educational level was classified as no formal education, sishu/homeschool/elementary school, and middle school and above. Marital status was combined into two groups, cohabited and living alone. Smoking status is a binary variable (yes or no). Sleep duration was identified according to the self-reported answer of “During the past month, how many hours of actual sleep did you get at night (average hours for one night)?” [27]. Participants were asked “During the past month, how long (minutes) did you take a nap after lunch?”, based on which the daytime napping duration was recognized. BMI was calculated as weight (kg) divided by height squared (m^2^). Handgrip strength was recommended to represent the skeletal muscle strength according to AWGS 2019. The standard device was used to examine the handgrip strength, with the low HGS cut-off points < 18 kg and < 28 kg for women and men respectively [28]. In CHARLS, handgrip strength was measured twice for each hand, and the maximum grip strength of the accustomed hand was taken as the grip strength value. IADL was evaluated based on individuals’ ability to do daily housework, make a telephone call, cook, take medicine, go shopping, and manage finances. Each answer was divided into four responses, as follows: “can do it by myself”, “have some difficulties”, “need help” and “cannot do it”. The elderly who had any difficulty in any item were classified as dependent. The pain was assessed using the question “Do you feel any pain?” and was classified into two categories. Comorbidities were assessed as the number of chronic diseases categorized as none, one, and more than one. Particularly, chronic diseases including hypertension, dyslipidemia, diabetes mellitus, stroke, cancer, chronic lung diseases, liver disease, heart attack, kidney disease, digestive disease, emotional problems, memory-related disease, asthma, and arthritis were identified based on the answer of the question “Have you been diagnosed with conditions listed below by a doctor” with a “yes” or “no” answer. Depression was assessed by the 10-item Center for Epidemiologic Studies Depression Scale (CES-D 10) [29].

### Statistical analysis

Data are reported as number (%) for categorical variables and mean (standard deviation) for continuous variables. In cross-sectional analysis, multivariate logistic regression was utilized to estimate the association of usual alcohol consumption with baseline fall (yes or no), adjusting for age, gender, IADL, pain, education, residence, marital status, comorbidities, handgrip strength, smoking status, depression, self-reported sleep duration, daytime napping, BMI. The 95% confidence intervals (CIs) were calculated for odds ratio estimates across different categories. In addition, we evaluated the dose–response relationship between usual alcohol consumption, as continuous change, and the risk of falling using restricted cubic splines [30]. The nonlinear trend was considered to exist if the second order term *P* < 0.05, which was assessed using the Wald Chi-square tests. In prospective analyses, falling risk during 3 years of follow-up was evaluated too using multivariable logistic regression. The dose–response relationship was also examined. Tolerance and variance inflation factor were calculated to assess whether the collinearity existed. The tolerance was > 0.1, and variance inflation factor did not exceed > 5.0, denoting that there was no collinearity among them [31].

Sensitivity analyses were also conducted. 1) in the prospective analysis, an additional analysis that excluded participants that reported at least a fall at baseline was conducted; 2) we excluded former drinkers and reanalyzed the dose–response relationship between alcohol consumption and risk of fall to test the robustness. All statistical analysis was performed using Stata version 16.0, SAS version 9.4, and R version 4.1.0. Two-sided *P* < 0.05 was considered statistically significant.

## Results

Table 1 showed the characteristics of participants in the different categories of usual alcohol consumption status in the longitudinal analysis. Overall, the proportion of males was 46.0% among all participants (*n* = 5371). The mean age of the participants was 61.0 years and 6380 participants (54.7%) were aged 60 years or above. Of 11,667 middle-aged and older participants, more than half (65.0%) of the participants were never drinkers, and 26.0% reported that they had drunk more than once a month, including 15.1% had moderate alcohol consumption and 10.9% had excessive alcohol consumption.

**Fig. 1:**
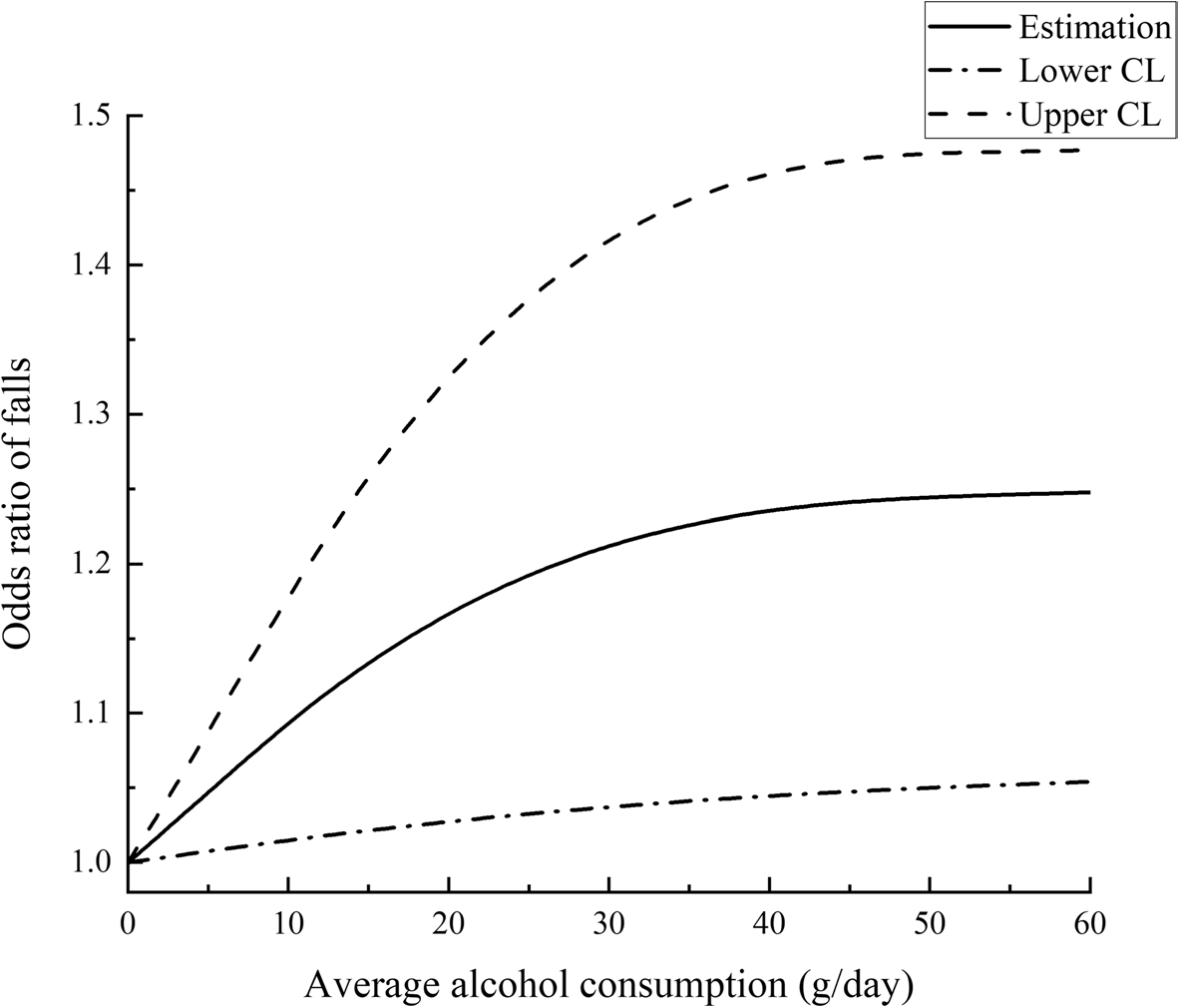
Odds ratios and corresponding 95% confidence intervals describing the dose–response relationship between alcohol consumption and 3-year risk of falls among middle-aged and older Chinese adults (Adjusted for all variables in Table 1). Alcohol consumption of 0 g/day was used as the reference

Alcohol consumption was likely to increase the risk of falls among middle-aged and older Chinese adults in longitudinal analysis. Former drinkers, moderate drinkers, and excessive drinkers were more likely to report falling than never drinkers (former: OR, 1.24; 95% CI, 1.05–1.46; moderate: OR, 1.22; 95% CI, 1.06–1.41; excessive: OR, 1.36; 95% CI, 1.15–1.61) (Table 2). We also used restricted cubic splines to estimate the trend in the risk for falls. The spline function for usual alcohol consumption confirmed the nonlinear relationship with the risk of falls (the second order term *P* = 0.0317), which was shown in Fig. 1. The spline function for usual alcohol consumption confirmed the nonlinear relationship with the risk of falls, which was shown in Fig. 1. Apparently, with increasing usual alcohol consumption, the risk of falls was on the rise. Compared to 0 g/day, the risk of fall with alcohol consumption was near-linearly associated up to 40 g/day, where the OR is 1.24(1.04,1.46) (Table 3). The dose–response curve went up slowly thereafter.

**Table 2:** Multivariable logistic regression on usual alcohol consumption and three-year risk of self-reported falls (Model 1) and baseline falls (Model 2)

Variables	Model 1 (*n* = 11,667)	Model 2 (*n* = 12,910)
Age, years	**1.02(1.01,1.02)**	**1.01(1.01,1.02)**
Gender		
Female	1	1
Male	0.66(0.58,0.76)	0.74(0.65,0.84)
Residence		
Urban	1	1
Rural	1.00(0.90,1.10)	1.04(0.94,1.16)
Education		
No formal education	1	1
Sishu/Homeschool/Elementary school	0.95(0.84,1.06)	1.00(0.89,1.12)
Middle school and above	0.95(0.83,1.10)	0.95(0.82,1.10)
Marital status		
Cohabited	1	1
Living alone	**1.21(1.07,1.36)**	1.07(0.95,1.20)
Smoking status		
No	1	1
Yes	1.06(0.93,1.20)	1.03(0.91,1.18)
Sleep duration		
< 7 h	**1.19(1.04,1.36)**	**1.19(1.04,1.36)**
7–8 h	1	1
≥ 8 h	0.99(0.86,1.15)	0.95(0.81,1.10)
Midday nap		
0 min	1	1
0–30 min	1.03(0.90,1.18)	0.99(0.86,1.14)
30–60 min	0.93(0.80,1.09)	0.91(0.78,1.06)
> 60 min	0.89(0.76,1.05)	0.90(0.77,1.07)
Body mass index		
Underweight	0.98(0.80,1.18)	1.12(0.92,1.36)
Normal	1	1
Overweight	0.91(0.75,1.12)	1.16(0.94,1.42)
Obesity	0.93(0.74,1.16)	1.05(0.83,1.32)
Depression		
No	1	1
Yes	**1.27(1.15,1.4)**	**1.37(1.24,1.52)**
IADL ^a^		
Independent	1	1
Dependent	**1.25(1.09,1.42)**	**1.40(1.23,1.59)**
Pain		
No	1	1
Yes	**1.39(1.26,1.55)**	**1.77(1.59,1.96)**
Handgrip strength		
High	1	1
Low	**1.21(1.10,1.35)**	**1.28(1.15,1.42)**
Comorbidities		
0	1	1
1	1.13(0.99,1.27)	**1.20(1.05,1.36)**
≥ 2	**1.34(1.19,1.51)**	**1.47(1.30,1.66)**
Alcohol consumption		
Never drinkers	1	1
Former drinkers	**1.24(1.05,1.46)**	1.13(0.96,1.34)
Moderate drinkers	**1.22(1.06,1.41)**	**1.18(1.02,1.37)**
Excessive drinkers	**1.36(1.15,1.61)**	**1.32(1.11,1.57)**

^a^ *Abbreviations*: *IADL* instrumental activities of daily living

^b^ Bold values indicate *P* < 0.05

^c^ The model adjusted for all variables in Table 1 including age, gender, IADL, pain, education, residence, marital status, comorbidities, handgrip strength, smoking status, depression, self-reported sleep duration, daytime napping, BMI

**Table 3:** Risk of falls associated with alcohol consumption level

alcohol consumption (g/day)	ORs(95%CIs) in longitudinal analysis (*n* = 11,667)	ORs(95%CIs) in cross-sectional analysis (*n* = 12,910)
0	Ref	Ref
5	**1.05(1.01,1.09)**	**1.03(1.00,1.06)**
10	**1.09(1.01,1.18)**	**1.06(1.01,1.12)**
20	**1.17(1.03,1.32)**	**1.13(1.02,1.26)**
30	**1.21(1.04,1.42)**	**1.19(1.02,1.38)**
40	**1.24(1.04,1.46)**	**1.22(1.03,1.45)**
50	**1.24(1.05,1.47)**	**1.24(1.03,1.48)**

^a^ Bold values indicate *P* < 0.05

^b^ The model adjusted for all variables in Table 1 including age, gender, IADL, pain, education, residence, marital status, comorbidities, handgrip strength, smoking status, depression, self-reported sleep duration, daytime napping, BMI

**Fig. 2:**
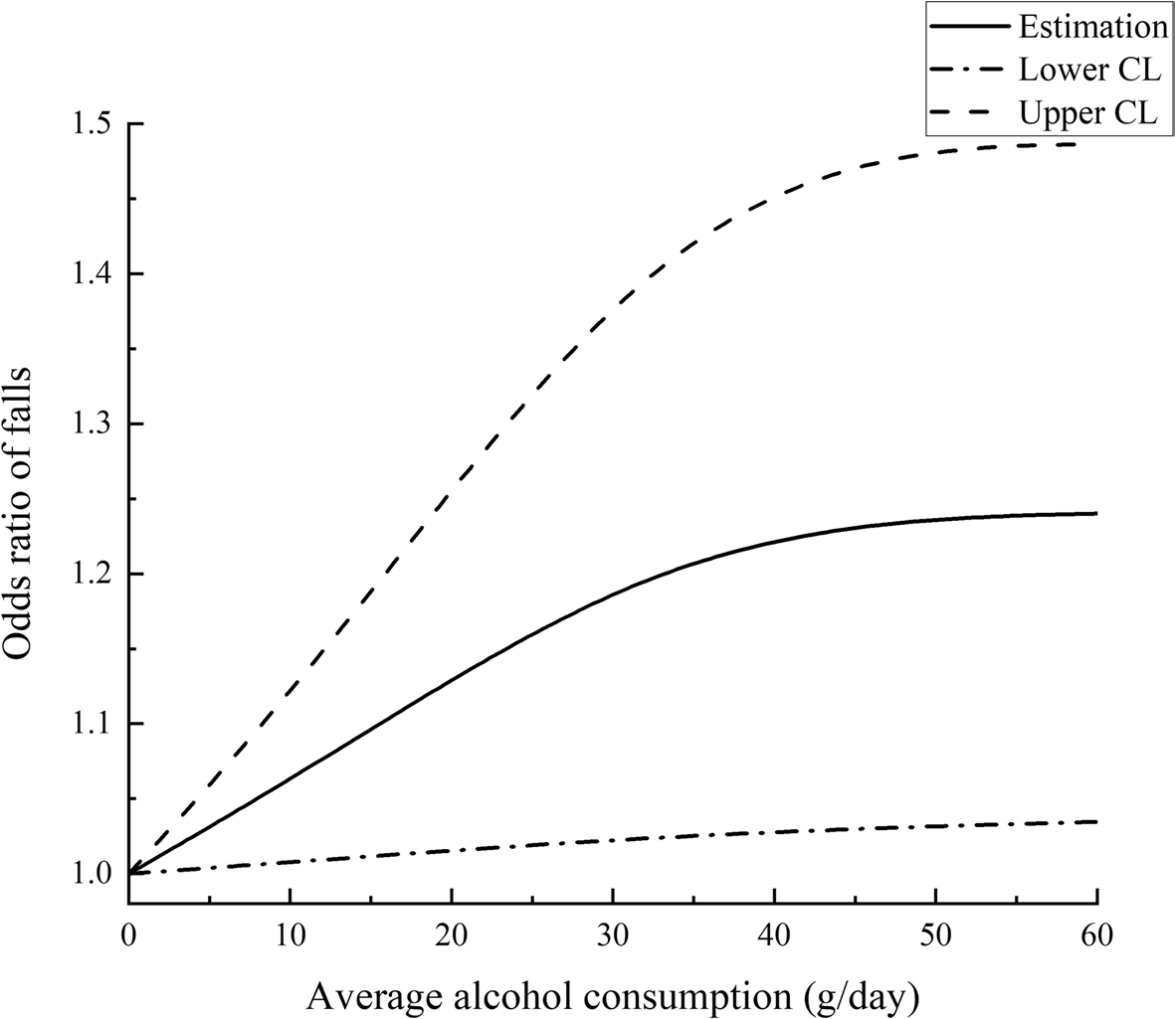
Odds ratios and corresponding 95% confidence intervals describing the dose–response relationship between alcohol consumption and baseline falls risk among middle-aged and older Chinese adults (Adjusted for all variables in Table 1). Alcohol consumption of 0 g/day was used as the reference

Results both from logistic model and dose–response analysis in cross-sectional analyses bore an extreme resemblance to the prospective analyses. Moderate drinkers and excessive drinkers were more likely to report falling than never-drinkers (moderate: OR, 1.18; 95% CI, 1.02–1.37; excessive: OR, 1.32; 95% CI, 1.11,1.57) (Table 2). However, compared with never drinkers, we reported no significant association of falling with former drinkers (former: OR, 1.13; 95% CI, 0.96–1.34) (Table 2). The association of usual alcohol consumption with risk of 3-year fall followed a significantly nonlinear trend(the second order term *P* = 0.0354), appeared to rise quickly up to 40 g/day and from then was upward slowly (Fig. 2). The specific figure can be obtained from Table 3, for example, the fall risk of 30 g/day drinkers was 1.19(1.02,1.38) in the cross-sectional analysis compared to 0 g/day. The results of collinearity tests showed that there was no collinearity between the variables (Supplementary Table S2).

### Sensitivity analysis

The sensitivity analysis that excluded participants who reported falling at baseline yielded similar results to the cross-sectional analysis. Moderate drinkers and excessive drinkers were at higher risk of falls compared to never drinkers (moderate: OR, 1.26; 95% CI, 1.06–1.49; excessive: OR, 1.41; 95% CI, 1.15–1.72). No significant increased risk of falls was found for former drinkers (former: OR, 1.22; 95% CI, 1.00–1.50) (Supplementary Table S1), with a *P* value of 0.05. A significantly non-linear relationship was also found, with a quite analogous shape of the curve to our main results (the second order term *P* = 0.0182) (Supplementary Figure S1). Additionally, significantly non-linear dose–response relationships were also consistent for both the prospective analysis (the second order term *P* = 0.0284) (Supplementary Figure S2) and the cross-sectional analysis (the second order term *P* = 0.0403) (Supplementary Figure S3) after excluding former drinkers.

## Discussion

Findings from the present study showed that individuals with moderate and excessive alcohol consumption were at increased risk of falls compared to never drinkers among middle-aged and older Chinese adults, both in cross-sectional and longitudinal analysis. However, compared to never-drinkers, former drinkers were more likely to encounter falls in the longitudinal analysis. Furthermore, we observed a significantly non-linear positive relationship between usual alcohol consumption and the risk of falls both cross-sectionally and longitudinally.

Falls risk of moderate drinkers and excessive drinkers was significantly higher than that of never-drinkers. Although in the cross-sectional study, the risk of falls in former drinkers is not statistically significant compared with never drinkers, we still consider that the risk of falls in former drinkers is greater than that in never drinkers. Because the directions of the two results are consistent, and we can see from Table 2 that the lower limit of the confidence interval is close to 1. Our sensitivity analysis also yielded similar conclusions, indicating the robustness of the results. Our results are consistent with previous studies that found that usual alcohol consumption was a significant risk factor for falls whether in young people or in the elderly [3–5]. Several mechanisms may account for the association of alcohol consumption with falls. First, it was reported that the intensity of fiber muscle atrophy and variation of ultrastructural were related to the ethanol quantity [32]. Second, long-time usual alcohol intake may generate cumulating adverse consequences and therefore lead to peripheral neuropathy, which was harmful to gait and balance, as well as bone health, resulting in skeletal muscle myopathy [16–18]. Third, excessive usual alcohol intake over the lifespan can increase the risk for cognitive decline and an increased risk for the development of dementia [21], which have been reported to increase the risk of falls in quite a few studies [19, 20, 22]. However, distinct conclusions that found no relationship between alcohol and falls also exist [7–9]. The possible reasons for the inconsistency are as follows: some studies used convenient sampling to select the participants, which might lead to selection bias [7]. The reference group of alcohol consumption in some studies was different from those in this study, and the covariates adjusted in the study might be insufficient because this study lacks some indicators that can represent the physical performance of participants, such as activity of daily living or grip strength, which have been proved to be crucial influencing factors of falls [14]. In addition, the low power of the test resulting from the small sample size might also explain the null association of some studies [8].

Inappropriate inclusion of sick quitters, who maintained a nondrinking status, in the reference group may result in inconsistent conclusions between the present study and others [8]. Protective effects or null associations may be confounded because those researchers failed to distinguish the lifetime abstainers and former drinkers [33, 34]. For instance, former drinkers have been reported by a large number of studies that they encounter poorer self-reported health and increased risk of mortality than never drinkers. Consequently, protective associations found among light drinkers may be less a consequence of a certain biological mechanism and more a statistical outcome resulting from the inappropriate categories of the reference group. Indeed, increasing research had found that the protective effect between alcohol consumption and specific health outcome was attenuated (*P* < 0.01) when former drinkers were excluded [35]. Such a finding suggests that effects may have been misestimated. Likewise, results from our study also agreed with this point. In our study, compared to lifetime abstainers, former drinkers were more likely to encounter falls. It will be more acceptable if the majority of former drinkers were previously excessive drinkers who have quit drinking for major health-related problems or differ on other critical features, which was also known as the sick quitter phenomenon [36].

To date, alcohol consumption studies in falls lack evaluation of any dose–response effects. Falls risk was on the rise incessantly as alcohol consumption increased, which was all along significantly higher than 1, indicating that there is no so-called “safe” alcohol consumption for falls. As a matter of fact, increasing literature had found that no alcohol intake reduced an individual’s risk the most [37, 38]. For instance, findings from a large school-based survey about alcohol carried out in Hong Kong suggested that even light alcohol intake was associated with depressive symptoms [39]. By quantifying the risk for nonfatal fall injuries associated with alcohol intake, our findings highlight the significance of obeying the recommendations for alcohol abstinence. Necessary assessment and intervention programs are supposed to be developed to prevent and reduce fall injuries as much as possible among middle-aged and older people who engage in drinking.

Several causes might account for the dose–response trend, with the overall risk increasing across drinking quantities but in a decelerating manner. First, this visual trend of an observed declined rising rate in risk may be genuine (e.g., assuming that one may be too incapable to encounter a fall when at pretty large quantities of alcohol intake) [40]. Second, perhaps individuals who have higher alcohol intake tend to more likely consume specific beverages which are not associated with falls risk [41]. Liquor and beer are the most common beverages in China, as in our study accounted for 72.55% and 41.65% of all current drinkers respectively, while wine or rice wine only 19.28%. It is noteworthy that Samuel et al. [42] found that the climbing speed of the curves between spirits, beer, and cider alcohol intake and incident atrial fibrillation declined along with the alcohol quantities, which was contrary to red wine and white wine, indicating the potential effect of type of beverages on the dose–response curve. Third, in consideration of the scarce data available at higher alcohol intake levels, this might due to an artefact rather than any real mechanism [43]. Finally, heavy drinkers may under-report their real alcohol intake, and this would lead to the underestimation of any true association, which might yield a flatter curve [44].

There are a number of strengths to our study. We retrospectively and prospectively evaluated associations to guarantee the robustness of the results. Besides, we utilized two measures of alcohol consumption in an attempt to comprehensively capture its association with the risk of falls. The population-representative design of the CHARLS study, the large number of middle-aged and older participants, and the high overall response in the CHARLS study also enhanced the results of our study. Nevertheless, several limitations should be recognized. Subjects with missing data on covariates were excluded from analyses, which might generate bias in the results. Furthermore, information about usual alcohol consumption was obtained via a self-report scale, which might therefore introduce recall bias. Data on falls during the past 2 years were obtained via a self-report scale in CHARLS, which might introduce recall bias.

## Conclusions

In summary, our study suggests that usual alcohol consumption was significantly associated with a higher risk of falls compared with never drinkers both in the prospective analysis and retrospective analysis, showing a significantly non-linear relationship curve at the same time. These findings highlight the key role of alcohol intake on the risk of falls, which needed to be considered in developing effective intervention and prevention strategies for reducing falls among middle-aged and older Chinese adults.

## Supplementary Information

**Additional file 1:** **Table S1.** Multivariable logistic regression on usual alcohol consumption and three-year risk of self-reported falls among participants without baseline falls. **Table S2.** Collinearity tests in longitudinal analysis and cross-sectional analysis. **Figure S1.** Odds ratios and corresponding 95% confidence intervals describing the dose-response relationship between alcohol consumption and 3-year risk of falls among participants without baseline falls (Adjusted for all variables in Table 1). **FigureS2.** Odds ratios and corresponding 95% confidence intervals describing the dose-response relationship between alcohol consumption and 3-year risk of falls among middle-aged and older Chinese adults excluding former drinkers (Adjusted for all variablesin Table 1). **FigureS3.** Odds ratios and corresponding 95% confidence intervals describing the dose-response relationship between alcohol consumption and baseline falls risk among middle-aged and older Chinese adults excluding former drinkers (Adjusted for all variablesin Table 1).

## Data Availability

The datasets supporting the conclusions of this article are available publicly, http://charls.pku.edu.cn/pages/data/111/en.html.

## Abbreviations

IADL: Instrumental activities of daily living
BMI: Body Mass Index
CHARLS: Chinese Health and Retirement Longitudinal Study
CIs: Confidence intervals
ORs: Odds ratios
