# Supplementary material for: Association between usual alcohol consumption and risk of falls in middle-aged and older Chinese adults
Source: BMC Geriatr. 2022 Sep 14;22:750. doi: 10.1186/s12877-022-03429-1 (PMC9472419; doi:10.1186/s12877-022-03429-1)
Supplement: Supplementary file 1 — Additional file 1: Table S1. Multivariable logistic regression on usual alcohol consumption and three-year risk of self-reported falls among participants without baseline falls. Table S2. Collinearity tests in longitudinal analysis and cross-sectional analysis. Figure S1. Odds ratios and corresponding 95% confidence intervals describing the dose-response relationship between alcohol consumption and 3-year risk of falls among participants without baseline falls (Adjusted for all variables in Table 1). FigureS2. Odds ratios and corresponding 95% confidence intervals describing the dose-response relationship between alcohol consumption and 3-year risk of falls among middle-aged and older Chinese adults excluding former drinkers (Adjusted for all variablesin Table 1). FigureS3. Odds ratios and corresponding 95% confidence intervals describing the dose-response relationship between alcohol consumption and baseline falls risk among middle-aged and older Chinese adults excluding former drinkers (Adjusted for all variablesin Table 1). [file 12877_2022_3429_MOESM1_ESM.docx]

**Additional file**

Table S1 Multivariable Logistic Regression on Usual Alcohol Consumption and Three-Year Risk of Self-Reported Falls among Participants without Baseline Falls

| Variables | ORs(95%CI) |
| --- | --- |
| Age, years | **1.01(1.01,1.02)** |
| Gender | |
| Female | 1 |
| Male | **0.70(0.60,0.82)** |
| Residence | |
| Urban | 1 |
| Rural | 1.01(0.89,1.13) |
| Education | |
| No formal education | 1 |
| Sishu/Homeschool/Elementary school | 0.96(0.83,1.10) |
| Middle school and above | 0.96(0.81,1.13) |
| Marital status | |
| Cohabited | 1 |
| Living alone | **1.26(1.09,1.45)** |
| Smoking status | |
| No | 1 |
| Yes | 1.05(0.90,1.22) |
| Sleep duration | |
| <7h | **1.18(1.01,1.38)** |
| 7-8h | 1 |
| ≥8h | 0.98(0.82,1.16) |
| Midday nap | |
| 0 min | 1 |
| 0-30 min | 1.14(0.97,1.35) |
| 30-60 min | 1.02(0.85,1.23) |
| >60 min | 0.96(0.79,1.17) |
| Body mass index | |
| Underweight | 0.92(0.73,1.16) |
| Normal | 1 |
| Overweight | 0.81(0.64,1.04) |
| Obesity | 0.89(0.68,1.17) |
| Depression | |
| No | 1 |
| Yes | **1.19(1.05,1.34)** |
| IADL^a^ | |
| Independent | 1 |
| Dependent | 1.18(1.00,1.40) |
| Pain | |
| No | 1 |
| Yes | **1.27(1.12,1.45)** |
| Handgrip strength | |
| No | 1 |
| Yes | **1.18(1.04,1.34)** |
| Comorbidities | |
| 0 | 1 |
| 1 | 1.15(1.00,1.33) |
| ≥2 | **1.33(1.16,1.53)** |
| Alcohol consumption | |
| Never drinkers | 1 |
| Former drinkers | 1.22(1.00,1.50) |
| Moderate drinkers | **1.26(1.06,1.49)** |
| Excessive drinkers | **1.41(1.15,1.72)** |

^a^Abbreviations: IADL, instrumental activities of daily living.

^b^ Bold values indicate *P*<0.05.

^c^The model adjusted for all variables in Table 1 including age, gender, iadl, pain, education, residence, marital status, comorbidities, handgrip strength, smoking status, depression, self-reported sleep duration, daytime napping, BMI.

Table S2 Collinearity Tests in longitudinal analysis and cross-sectional analysis

| Variables | longitudinal analysis (n=11667) | | cross-sectional analysis (n=12910) | |
| --- | --- | --- | --- | --- |
|  | tolerance | variance inflation | tolerance | variance inflation |
| Age, years | 0.73834 | 1.35439 | 0.72337 | 1.38243 |
| Gender (Ref=Female) | | | | |
| Male | 0.51045 | 1.95906 | 0.51412 | 1.94509 |
| Residence (Ref=Urban) | | | | |
| Rural | 0.91927 | 1.08782 | 0.91442 | 1.09360 |
| Education (Ref=No formal education) | | | | |
| Sishu/Homeschool/ Elementary school | 0.58534 | 1.70840 | 0.58038 | 1.72301 |
| Middle school and above | 0.48930 | 2.04373 | 0.48442 | 2.06431 |
| Marital status (Ref=Cohabited) | | | | |
| Living alone | 0.93604 | 1.06833 | 0.92967 | 1.07565 |
| Smoking status (Ref=No) | | | | |
| Yes | 0.68213 | 1.46600 | 0.68562 | 1.45854 |
| Sleep duration (Ref=7-8h) | | | | |
| <7 h | 0.51769 | 1.93165 | 0.51251 | 1.95117 |
| ≥8 h | 0.52659 | 1.89901 | 0.52072 | 1.92040 |
| Midday nap (Ref=0 min) | | | | |
| 0-30 min | 0.85995 | 1.16286 | 0.85886 | 1.16434 |
| 30-60 min | 0.82497 | 1.21216 | 0.82407 | 1.21349 |
| > 60 min | 0.82978 | 1.20513 | 0.82871 | 1.20669 |
| Body mass index (Ref=Normal) | | | | |
| Underweight | 0.92921 | 1.07619 | 0.92219 | 1.08437 |
| Overweight | 0.84474 | 1.18380 | 0.84607 | 1.18193 |
| Obesity | 0.85075 | 1.17543 | 0.85371 | 1.17135 |
| Depression (Ref=No) | | | | |
| Yes | 0.85562 | 1.16874 | 0.85601 | 1.16822 |
| IADL^a^ (Ref=Independent) | | | | |
| Dependent | 0.88083 | 1.13530 | 0.86964 | 1.14990 |
| Pain (Ref=No) | | | | |
| Yes | 0.80619 | 1.24041 | 0.80409 | 1.24364 |
| Handgrip strength (Ref=High) | | | | |
| Low | 0.83561 | 1.19672 | 0.82248 | 1.21583 |
| Comorbidities (Ref=0) | | | | |
| 1 | 0.73329 | 1.36371 | 0.73564 | 1.35936 |
| ≥2 | 0.65213 | 1.53344 | 0.65358 | 1.53003 |
| Alcohol consumption (Ref=Never drinkers) | | | | |
| Former drinkers | 0.86642 | 1.15418 | 0.86439 | 1.15688 |
| Moderate drinkers | 0.81993 | 1.21962 | 0.81670 | 1.22443 |
| Excessive drinkers | 0.78108 | 1.28028 | 0.78015 | 1.28181 |

^a^Abbreviations: IADL, instrumental activities of daily living.


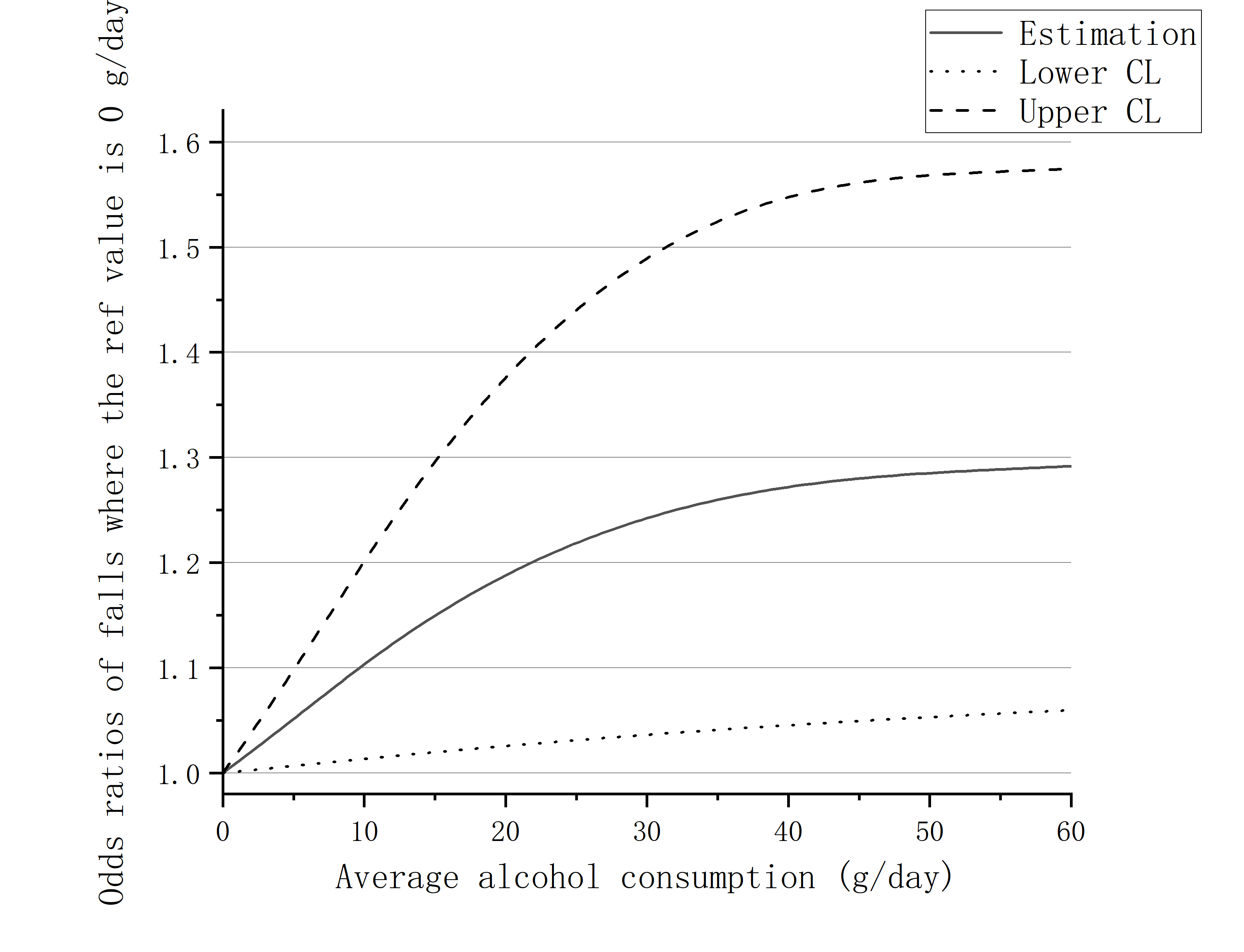


Figure S1 Odds Ratios and Corresponding 95% Confidence Intervals Describing the Dose-Response Relationship between Alcohol Consumption and 3-year Risk of Falls among Participants without Baseline Falls (Adjusted for all variables in Table 1).


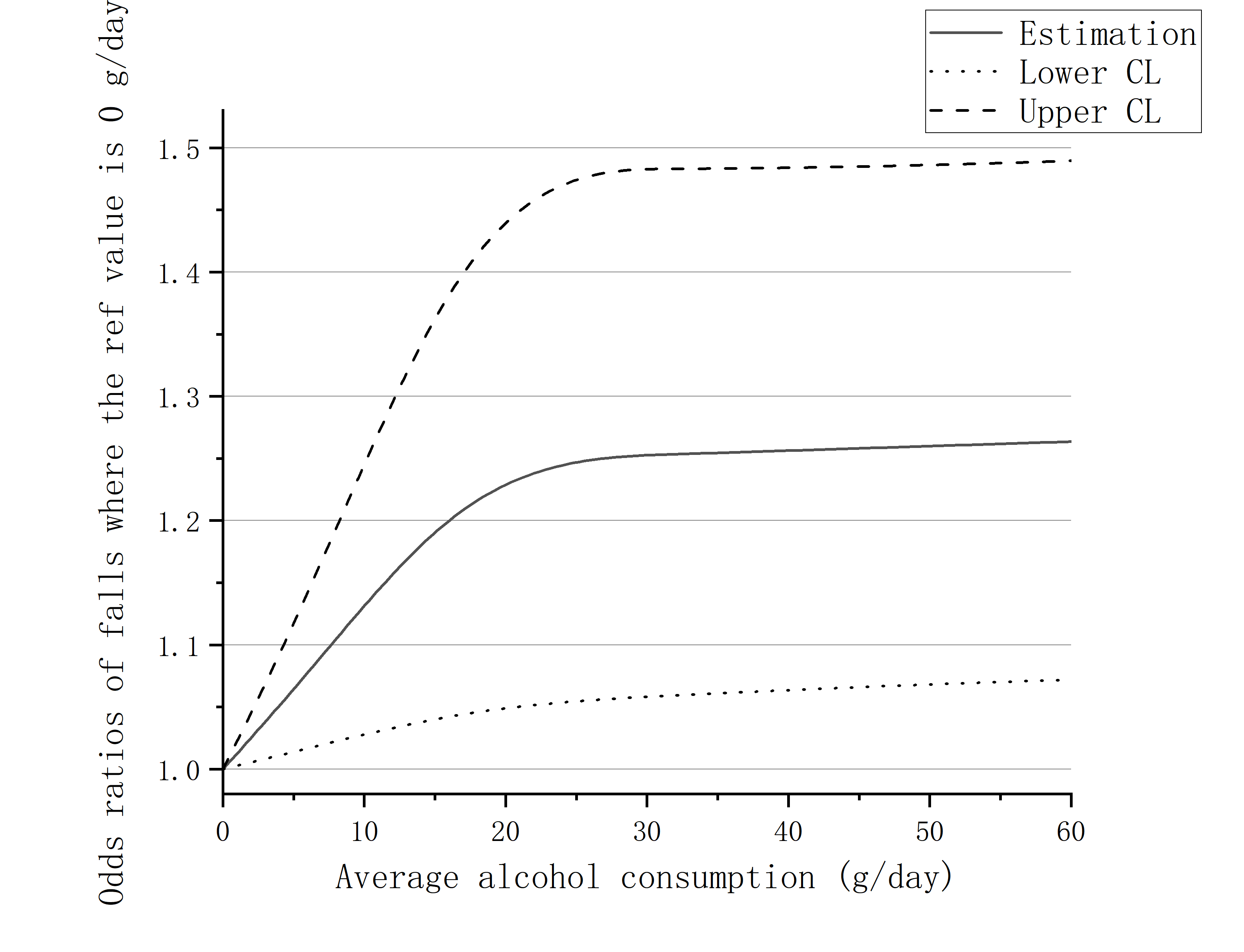


Figure S2 Odds Ratios and Corresponding 95% Confidence Intervals Describing the Dose-Response Relationship between Alcohol Consumption and 3-year Risk of Falls among Middle-aged and Older Chinese Adults Excluding Former Drinkers (Adjusted for all variables in Table 1).


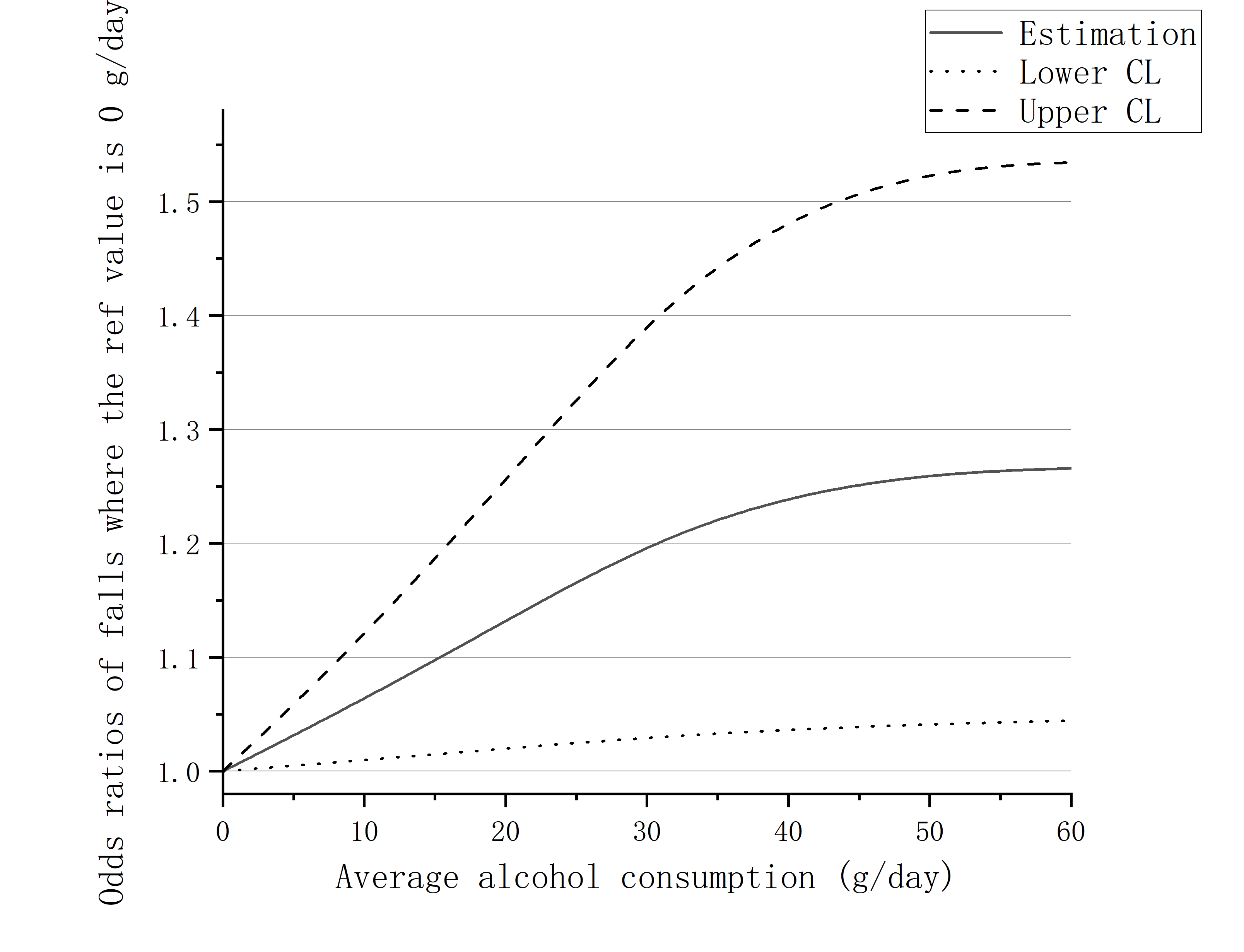


Figure S3 Odds Ratios and Corresponding 95% Confidence Intervals Describing the Dose-Response Relationship between Alcohol Consumption and Baseline Falls Risk among Middle-aged and Older Chinese Adults Excluding Former Drinkers (Adjusted for all variables in Table 1).
